# Supplementary material for: Protein stabilization of ITF2 by NF-κB prevents colitis-associated cancer development
Source: Nat Commun. 2023 Apr 25;14:2363. doi: 10.1038/s41467-023-38080-w (PMC10130090; doi:10.1038/s41467-023-38080-w)
Supplement: Supplementary file 1 — Supplementary Information [file 41467_2023_38080_MOESM1_ESM.pdf]

Nature communications

Online supplement

Protein Stabilization of ITF2 by NF-κB Prevents Colitis-associated Cancer Development

Authors: Mingyu Lee,[1,2,3,4,†] Yi-Sook Kim,[2,3,4,†] Suha Lim,[2,3,4] Seung-Hyun Shin,[5] Iljin Kim,[6] Jiyoung Kim,[2,4,7] Min Choi,[2,3,4] Jung Ho Kim,[8] Seong-Joon Koh,[9] Jong-Wan Park,[2,4,7] Hyun-Woo Shin,[2,3,4,7,10,11,*]

Supplementary Figure Legend

Lee et al, Fig. S1

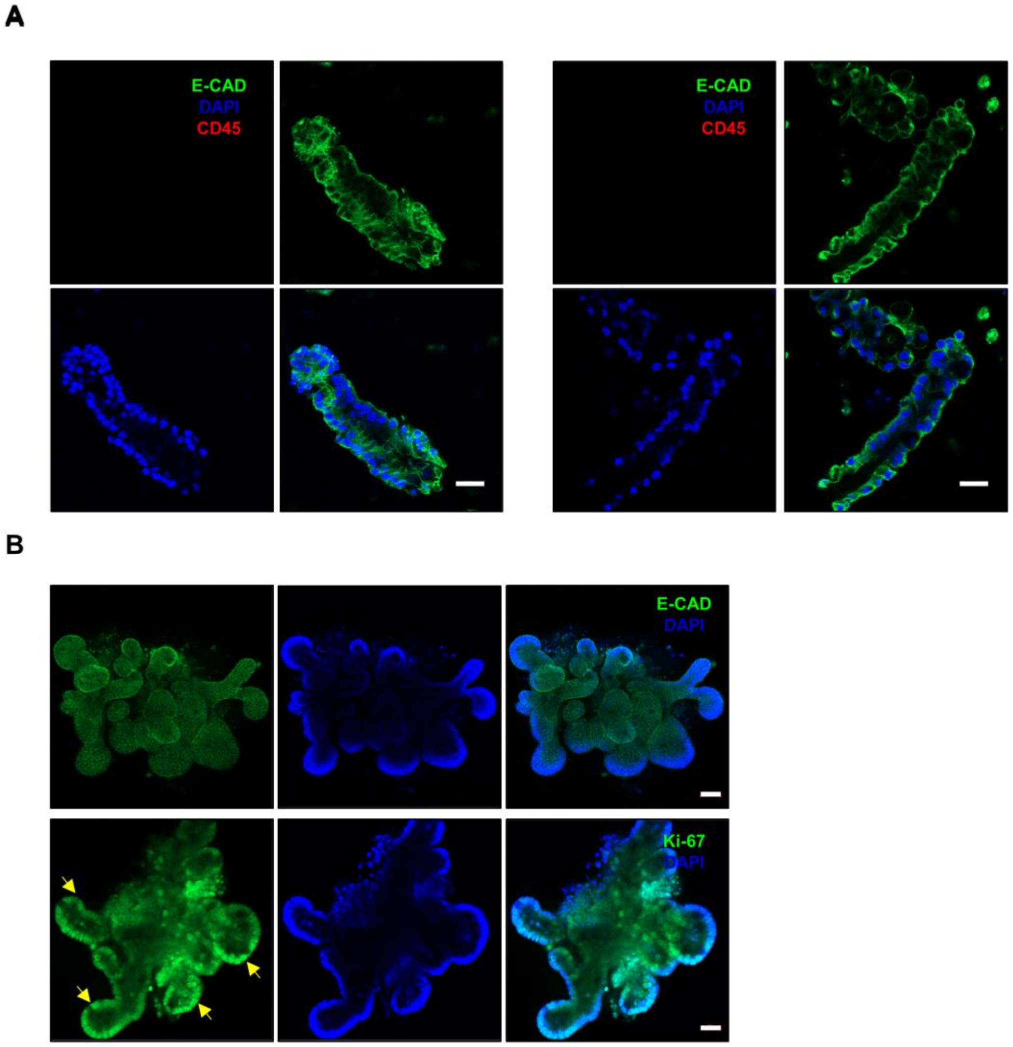

Figure S1. Immunofluorescence analysis of mouse crypt epithelial cells and mouse intestinal organoids. (A) Mouse crypt epithelial cells were isolated and stained with E-cadherin (green), CD45 (red), and DAPI. n=3, biologically independent experiment. **(B)** Mouse colonic organoids were stained with E-cadherin (green, top) or Ki-67 (green, bottom) with DAPI. Yellow arrows denoted Ki-67 positive cells. n=3, biologically independent experiment. Scale bars, 100 µm. Source data are provided in the Source Data file.

Lee et al, Fig. S2

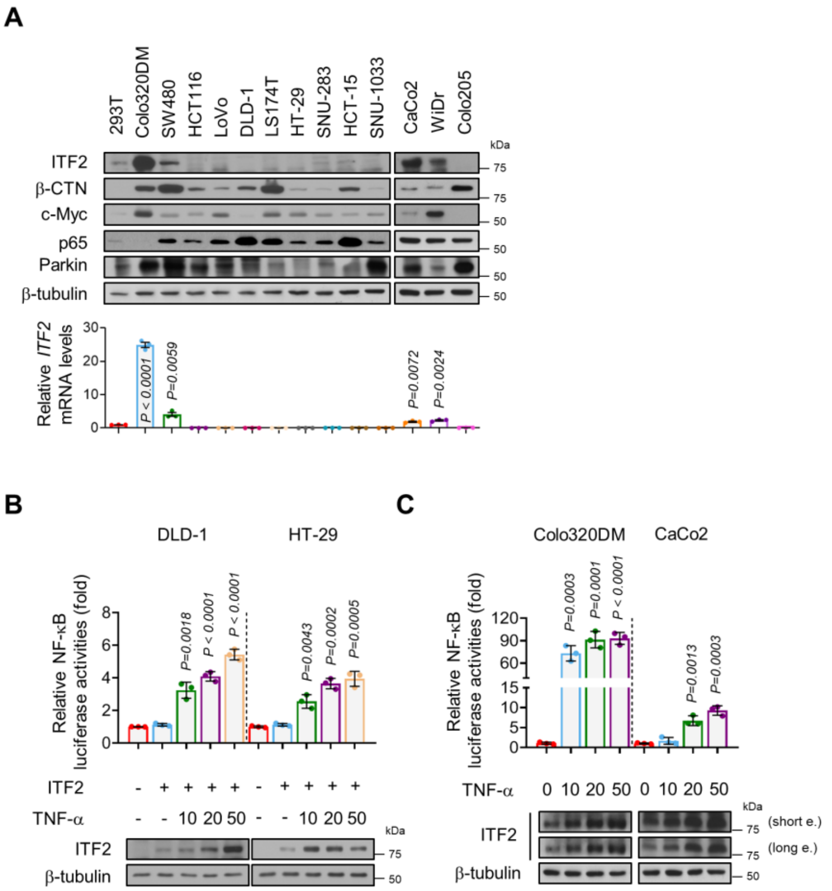

Figure S2. TNF-induced NF-κB activation promotes ITF2 expression in colon cancer cells. (A) HEK293T cells and colon cancer cell lines were collected for immunoblot and qRT-PCR. β-tubulin served as a loading control, and ITF2 mRNA expression was normalized by *GAPDH*. n=3, biologically independent experiment. **(B)** DLD1 and HT29 cells were co-transfected with κB-luciferase plasmid, CMV-β−gal plasmid, and pcITF2 plasmid followed by TNF treatment in a concentration-dependent manner for 8 h. pcDNA was used as a control vector. n=3, biologically independent experiment. **(C)** Colo320DM and CaCo2 cells were co-transfected with κB-luciferase plasmid and CMV-β−gal plasmid followed by TNF treatment in a concentration-dependent manner for 8 h. n=3, biologically independent experiment. Luciferase activity **(B, C)** was divided by β-gal activity. Results are denoted as relative values vs the control value and plotted. In all immunoblot analyses, the data are representative of three

independent experiments. All results are presented as means ± s.e.m. Statistical significance was determined by the unpaired two-tailed Student t-test **(A, B, C,** exact *P* values are shown in the figures**).** Source data are provided in the Source Data file.

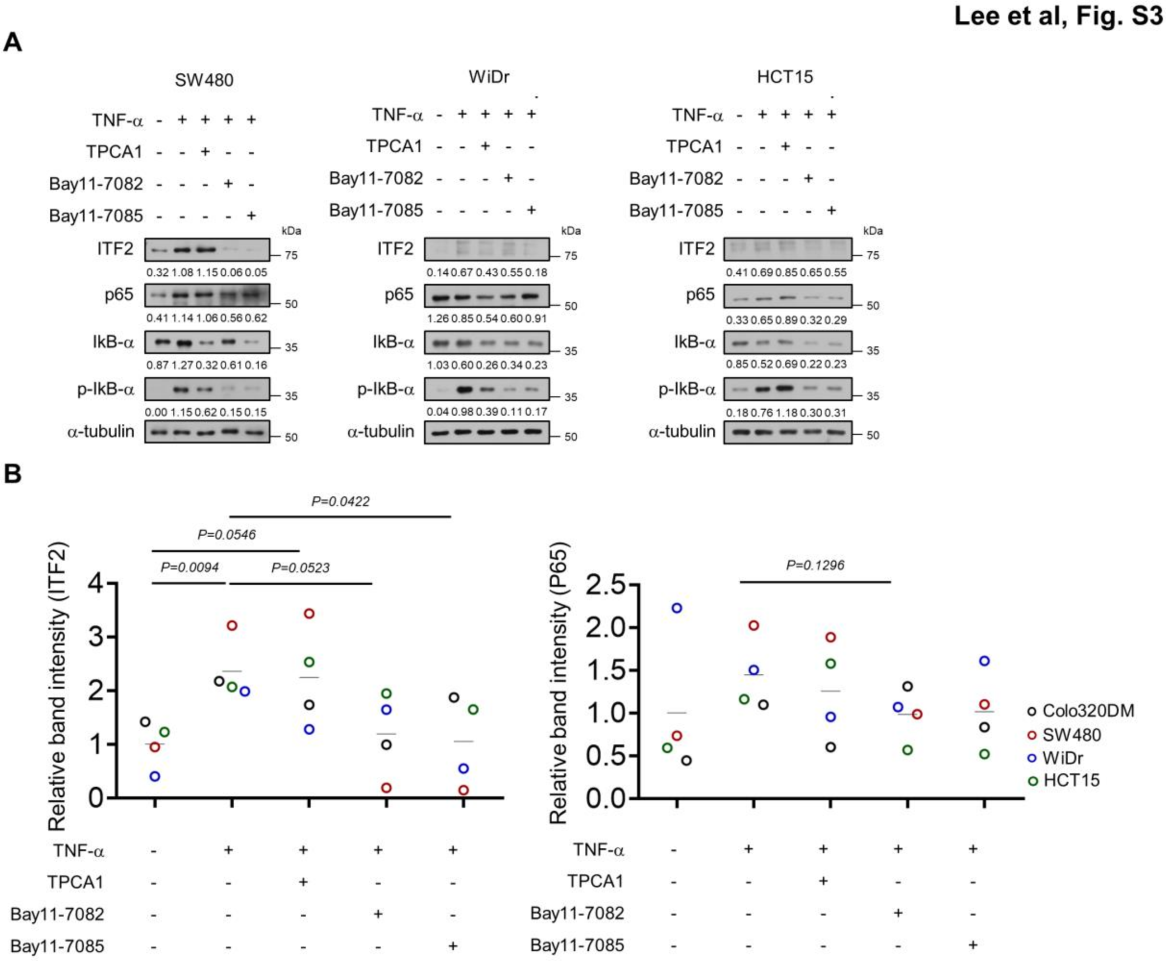

Figure S3. NF-κB is responsible for TNF-mediated ITF2 expression. (A, B) SW480, WiDr and HCT15 cells were pre-incubated with indicated inhibitors for 1 h followed by TNF stimulation for 8 h. The indicated proteins were traced and presented as immunoblots. Each data are expressed as normalized band intensity adjusted to α-tubulin, which serves as a loading control. In all immunoblot analyses, protein intensities were quantified by ImageJ software (n=3, biologically independent experiment, exact *P* values are shown in the figures). Statistical significance for normalized band intensity was determined by the unpaired two-tailed Student t-test **(B).** Data are shown as means ± s.e.m. Source data are provided in the Source Data file.

Lee et al, Fig. S4

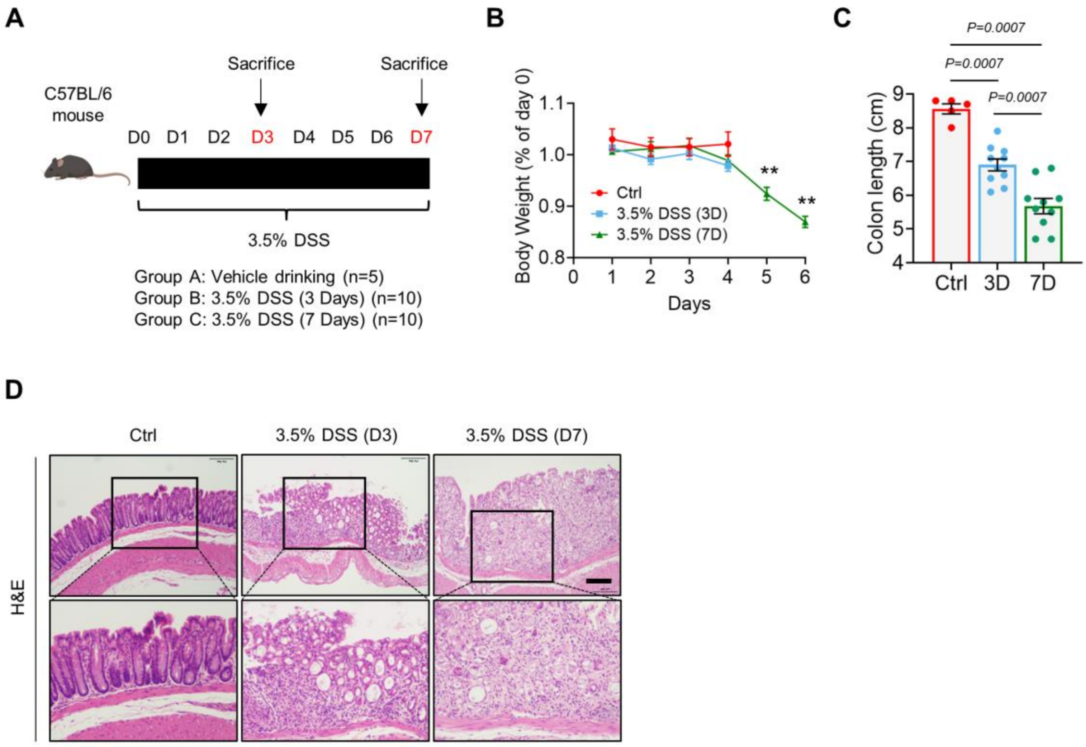

Figure S4. Features of acute colitis mice model. (A) Experimental design. C57BL/6 wild-type mice received 3.5% DSS in drinking water. On day3 and day7, all mice were sacrificed for further pathological analysis respectively. Mouse icon was created with BioRender (BioRender.com). **(B-D)** Body weight (**B,** 5w, $^{**}P = 0.0047$; 6w, $^{**}P = 0.0013$**)**, colon length (**C,** exact *P* values are shown in the figures) and representative H&E images **(D)** in mice treated with 3.5% DSS for 0-7 days (n=5, 10, 10 respectively, biologically independent animals for **B, C**). Scale bar, 100 μm. All results are presented as means ± s.e.m. Statistical significance was determined by Kruskal-Wallis tests followed by the two-tailed Mann–Whitney *U*-test **(B, C)** for pairwise comparisons. Source data are provided in the Source Data file.

Lee et al, Fig. S5

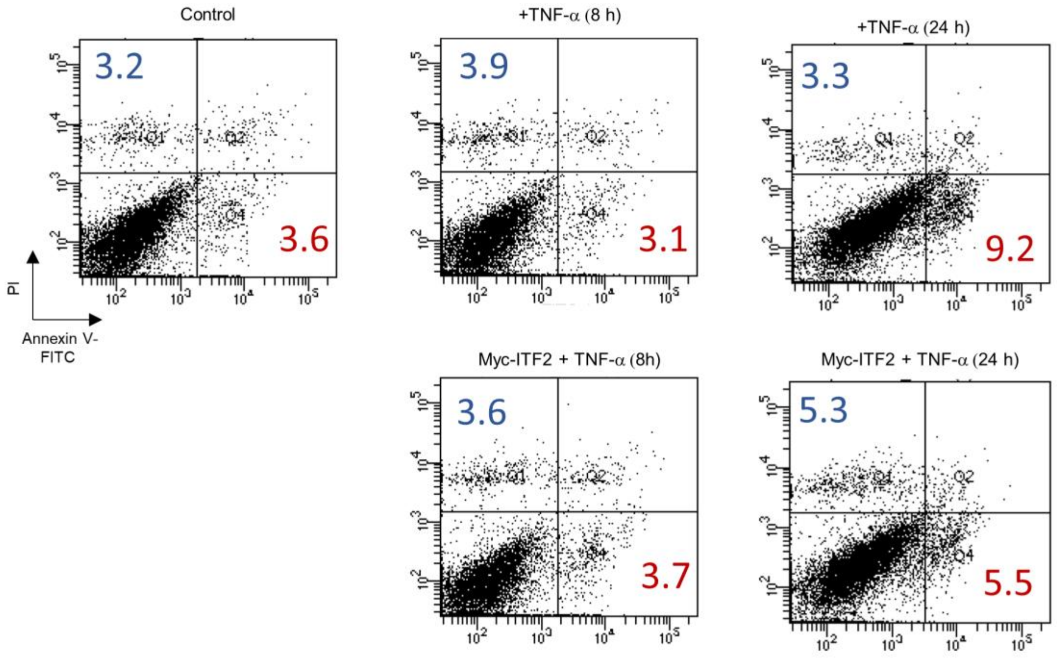

Figure S5. ITF2 has a minor effect on the TNF-induced cell death pathway. Representative FACS dot plots of Annexin V-FITC/PI staining results. The cells were transfected with Myc-ITF2 followed by TNF (20 ng/ml) for 8h and 24h respectively. The data are representative of three independent experiments. Source data are provided in the Source Data file.

Lee et al, Fig. S6

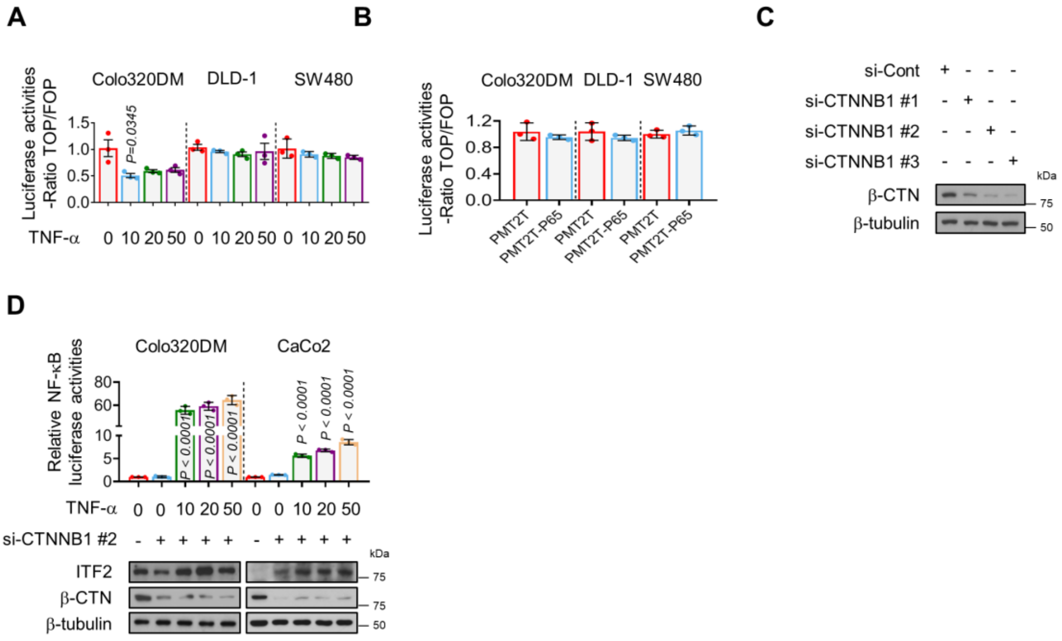

Figure S6. β-catenin has a minor effect on TNF-induced ITF2 expression. (A, B) The effect of TNF stimulation or p65 plasmid overexpression on β-catenin activation (n=3, biologically independent experiments). **(C)** Cells were subjected to siRNA knockdown targeting β-catenin (60 nM) for 48 h in Colo320DM cells, and indicated proteins were immunoblotted (n=3, biologically independent experiments). **(D)** Cells were transfected with siRNA targeting β-catenin followed by TNF challenge at a concentration-dependent manner for 8 h and indicated proteins were traced (n=3, biologically independent experiments). In all immunoblot analyses, the data are representative of three independent experiments. All results are presented as means ± s.e.m. Statistical significance was determined by the unpaired two-tailed Student t-test (**A, B, D,** exact *P* values are shown in the figures). Source data are provided in the Source Data file.

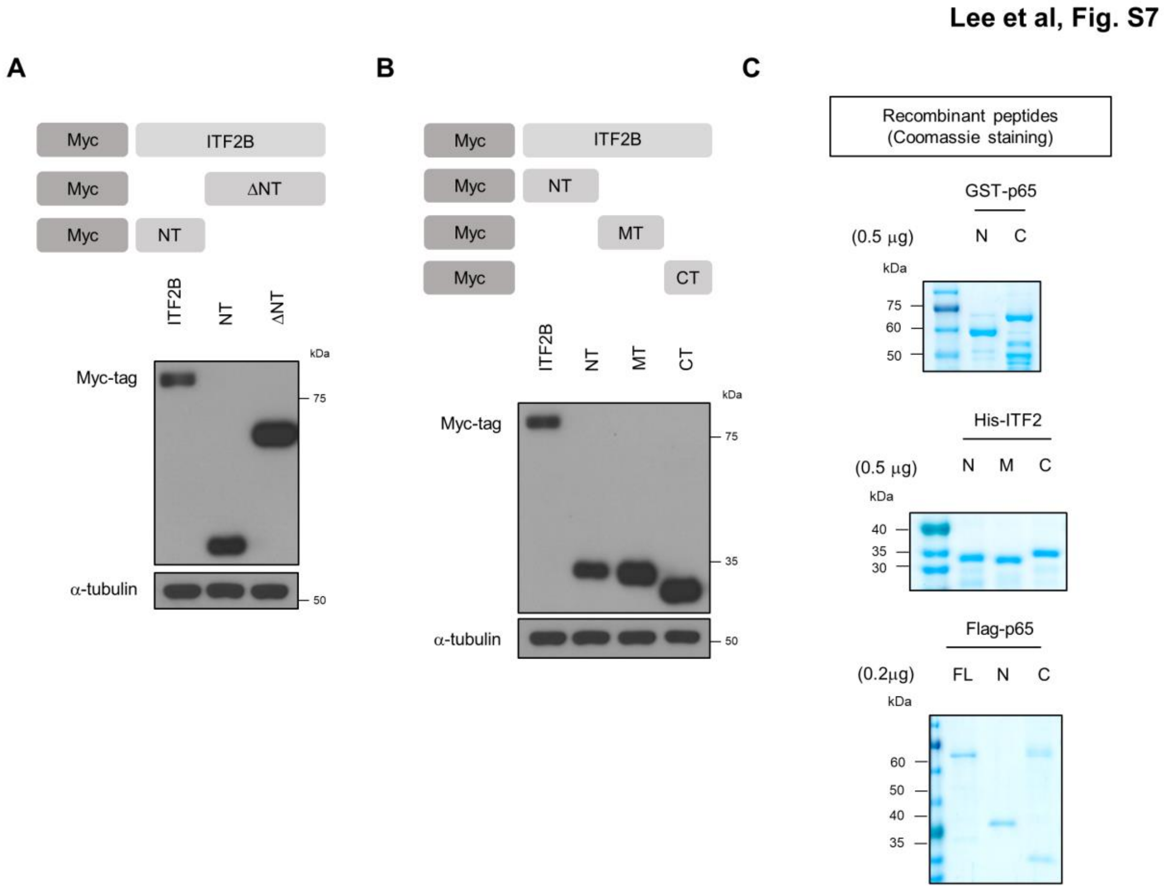

Figure S7. Validation of purified recombinant proteins. (A, B) HEK293T cells were transiently transfected with indicated plasmid vectors, and examined by immunoblotting (n=3, biologically independent experiments). **(C)** Recombinant GST-fused N-terminus (1–301, N), and C-terminus (302–551, C) peptides of P65 and His-tagged N-terminus (1–250, N), middle (251–500, M), and C-terminus (501–671, C) peptides of ITF2 were expressed in bacteria and purified using affinity beads. Each domain of Flag-65 proteins was obtained from the HEK293T cells. Purified peptides were electrophoresed and stained with Coomassie blue (n=3, biologically independent experiments). Source data are provided in the Source Data file.

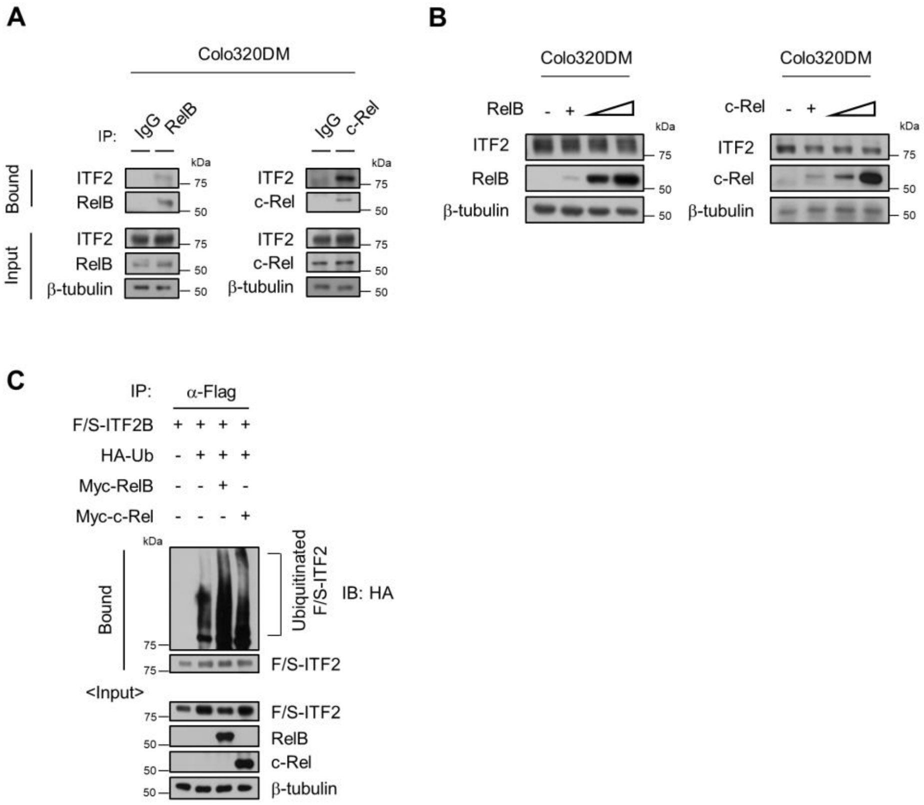

Figure S8. c-Rel and RelB are not crucial for TNF-induced ITF2 stabilization. (A) ITF2 interacts with RelB and c-Rel. Colo320DM cell lysates were immunoprecipitated with α-RelB or α-c-Rel, and immunoblotted. n=2, biologically independent experiments. **(B)** Colo320DM cells were transfected with either RelB or c-Rel in a dose-dependent manner, and then ITF2 expression was compared. n=2, biologically independent experiments **(C)** Plasmids encoding F/S-ITF2B were co-transfected with HA-Ub, Myc-RelB, and Myc-c-Rel into Colo320DM cells in the indicated combinations. The effects of the expression of RelB or c-Rel on ubiquitination of F/S-ITF2B were analyzed by using *in vivo* ubiquitination assays. n=2, biologically independent experiments. In all immunoblot analyses, the data are representative of three independent experiments. Source data are provided in the Source Data file.

Lee et al, Fig. S9

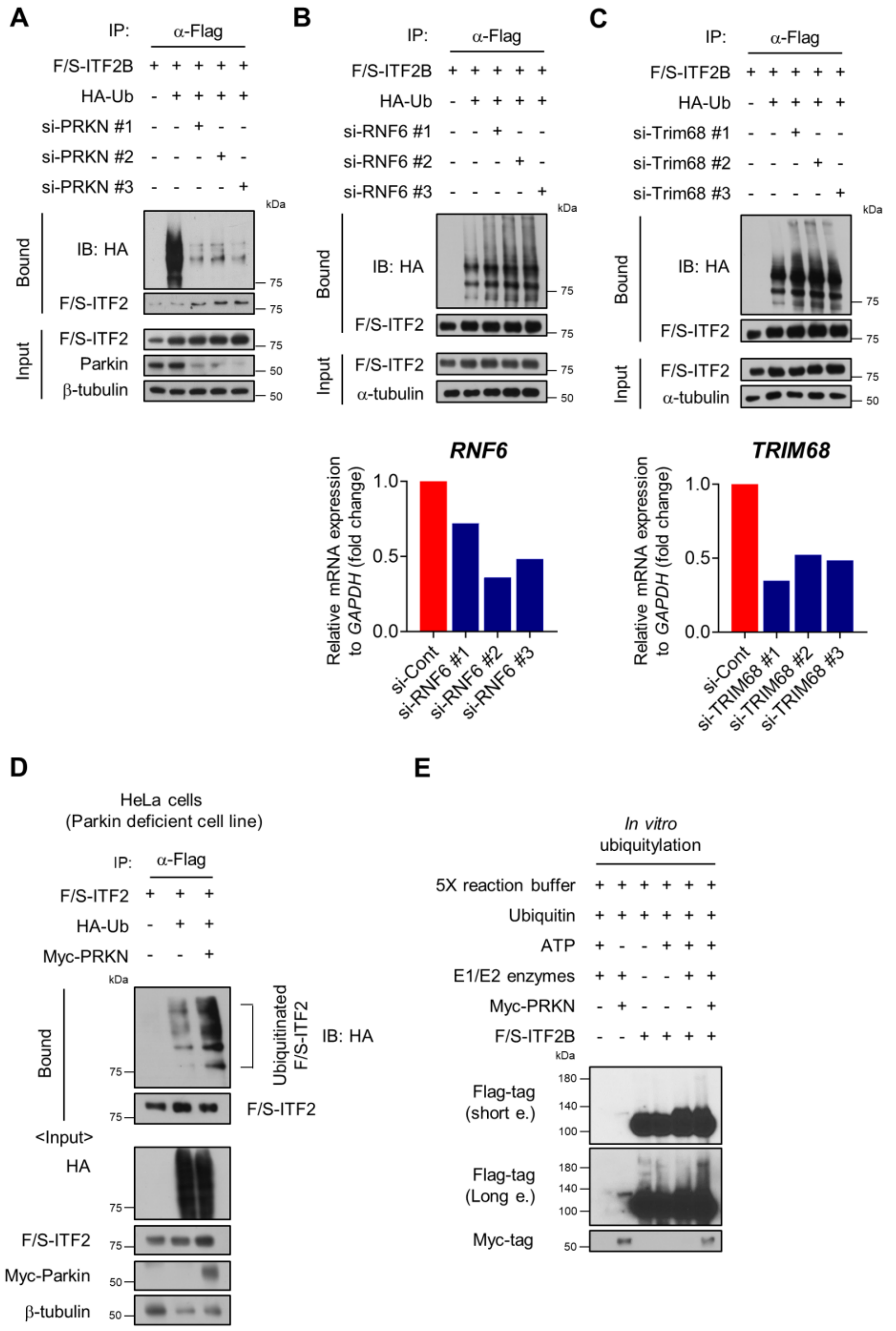

Figure S9. Parkin, not RNF6 and Trim68, is crucial for ubiquitin-mediated proteasomal degradation of ITF2. (A-C) Knockdown of endogenous Parkin decreased the ubiquitination of F/S-ITF2 in HEK293T cells. F/S-ITF2 was co-expressed with HA-Ub in HEK293T cells with or without siRNA targeting PRKN, RNF6, and Trim 68 respectively. n=3, biologically

independent experiments. q-PCR analysis was furtherly carried out to evaluate knockdown efficiency (bottom, n=1). **(D)** An experiment showed the effects of the expression of Myc-PRKN on the ubiquitination of F/S-ITF2 in HeLa cells. n=3, biologically independent experiments. **(E)** Parkin promoted ITF2 ubiquitination. For *in vitro* ubiquitination assays, Myc-Parkin, and F/S-ITF2B were eluted from HEK293 cells transfected with Myc-PRKN and F/S-ITF2B plasmids. The reactions were performed in the presence of the recombinant E1 and E2 enzymes as indicated and samples were immunoblotted with the indicated antibodies. n=3, biologically independent experiments. In all immunoblot analyses, the data are representative of three independent experiments. Source data are provided in the Source Data file.

Lee et al, Fig. S10

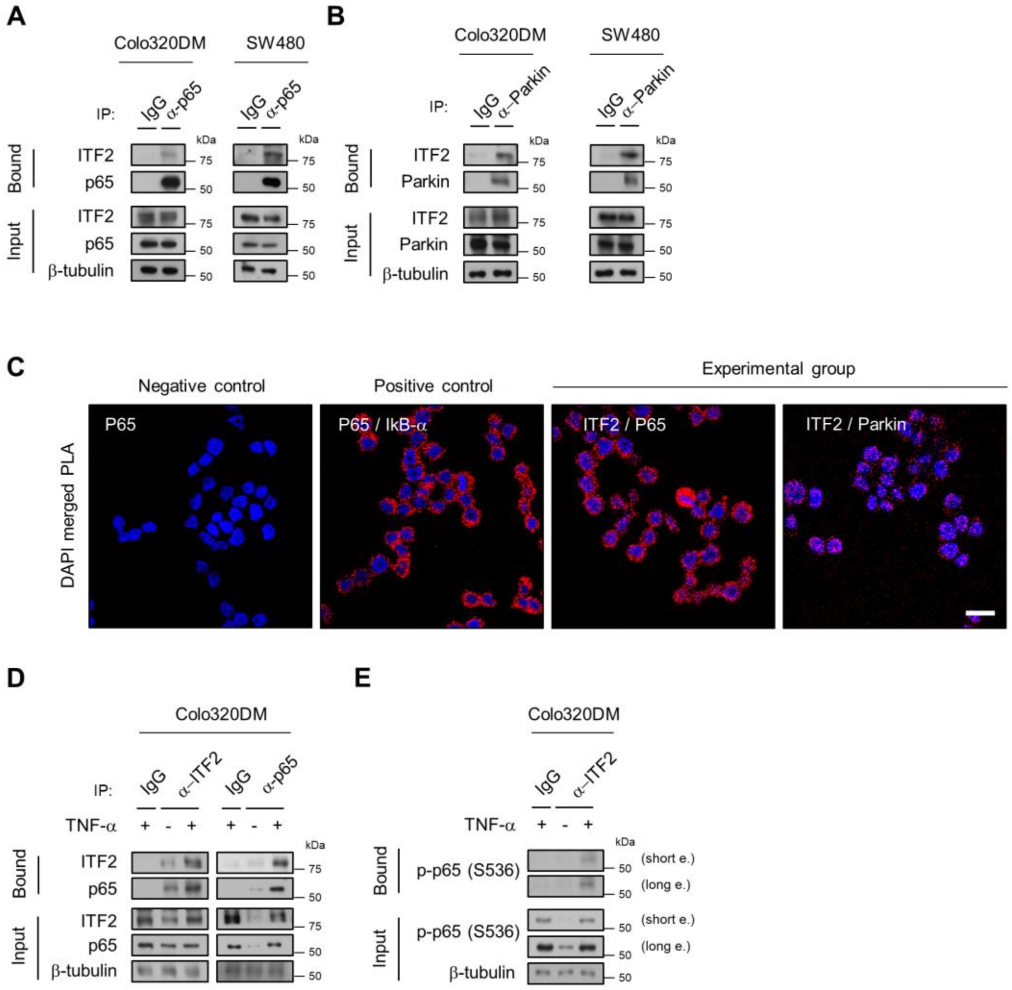

Figure S10. ITF2 interacts with p65 or Parkin in endogenous conditions. (A, B) ITF2 interacts with p65 or Parkin. Colo320DM and SW480 cell lysates were immunoprecipitated with α-p65 or α-Parkin, and immunoblotted. n=3, biologically independent experiments. **(C)** PLA assays on Colo320DM cells for the detection of proximity between ITF2 and other proteins (p65, Parkin) (n=3, biologically independent experiments). **(D, E)** Colo320DM cells were treated with TNF (20 ng/ml) for 8 h, and then immunoprecipitated with α-ITF2 or α-p65, and immunoblotted. n=3, biologically independent experiments. In all immunoblot analyses, the data are representative of three independent experiments. Scale bars, 100 μm. Source data are provided in the Source Data file.

Lee et al, Fig. S11

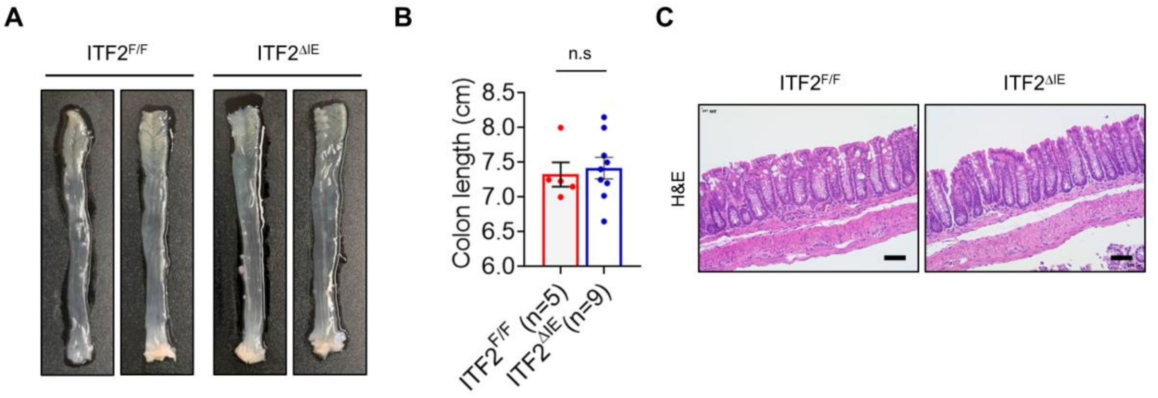

Figure S11. The effects of ITF2 knock out on colon tissues. Representative colon images **(A)**, colon length **(B)**, and H&E photographs **(C)** in *ITF2*$^{F/F}$ and *ITF2*$^{\Delta IE}$ mice (n=5, 9 respectively, biologically independent animals). Scale bar, 100 μm. All results are presented as means ± s.e.m. Statistical significance was determined by the two-tailed Mann–Whitney *U*-test for pairwise comparisons **(B).** NS, not significant. Source data are provided in the Source Data file.

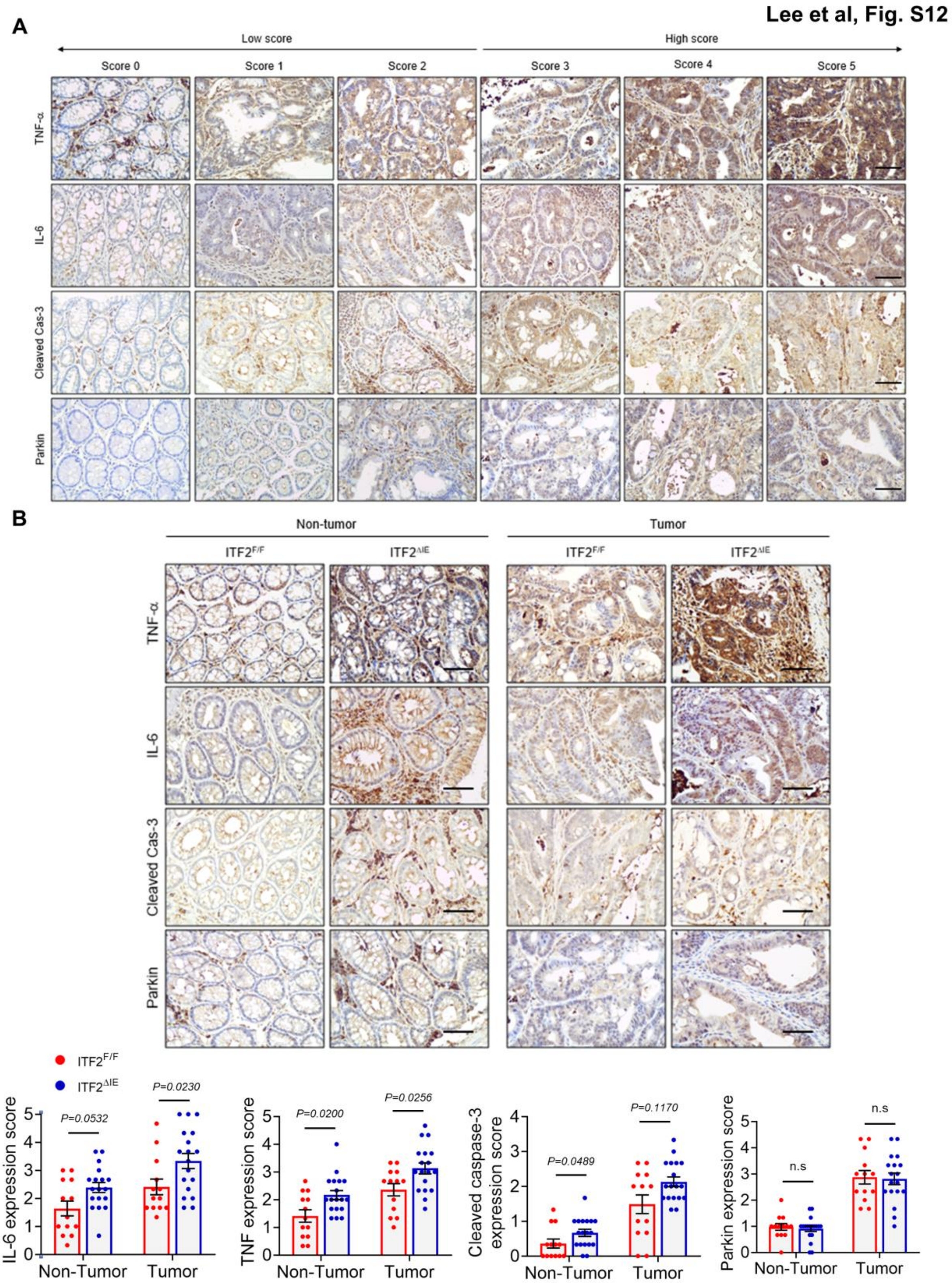

Figure S12. ITF2 loss increases NF-kB target gene expressions in AOM/DSS-challenged mice. (A)

Immunohistochemical (IHC) analysis of TNF-α, IL-6, Cleaved caspase-3, and Parkin expressions in AOM/DSS-treated mice tissues showing representative images of low (negative and intermediate) and high (moderate and strong) staining. Protein levels were estimated based on staining intensity (The data are representative of three independent experiments). **(B)** Representative images of IHC against indicated proteins (Top) and quantified results based on staining intensity (Bottom). Individual values are indicated by dots (*ITF2*$^{F/F}$, n=13; *ITF2*$^{\Delta IE}$, n=18, biologically independent animals). Scale bars, 100 μm. All results are presented as means ± SEM. Statistical significance was determined by the two-tailed Mann-Whitney *U* test for pairwise comparisons **(B,** exact *P* values are shown in the figures**).** NS, not significant. Source data are provided in the Source Data file.

Lee et al, Fig. S13

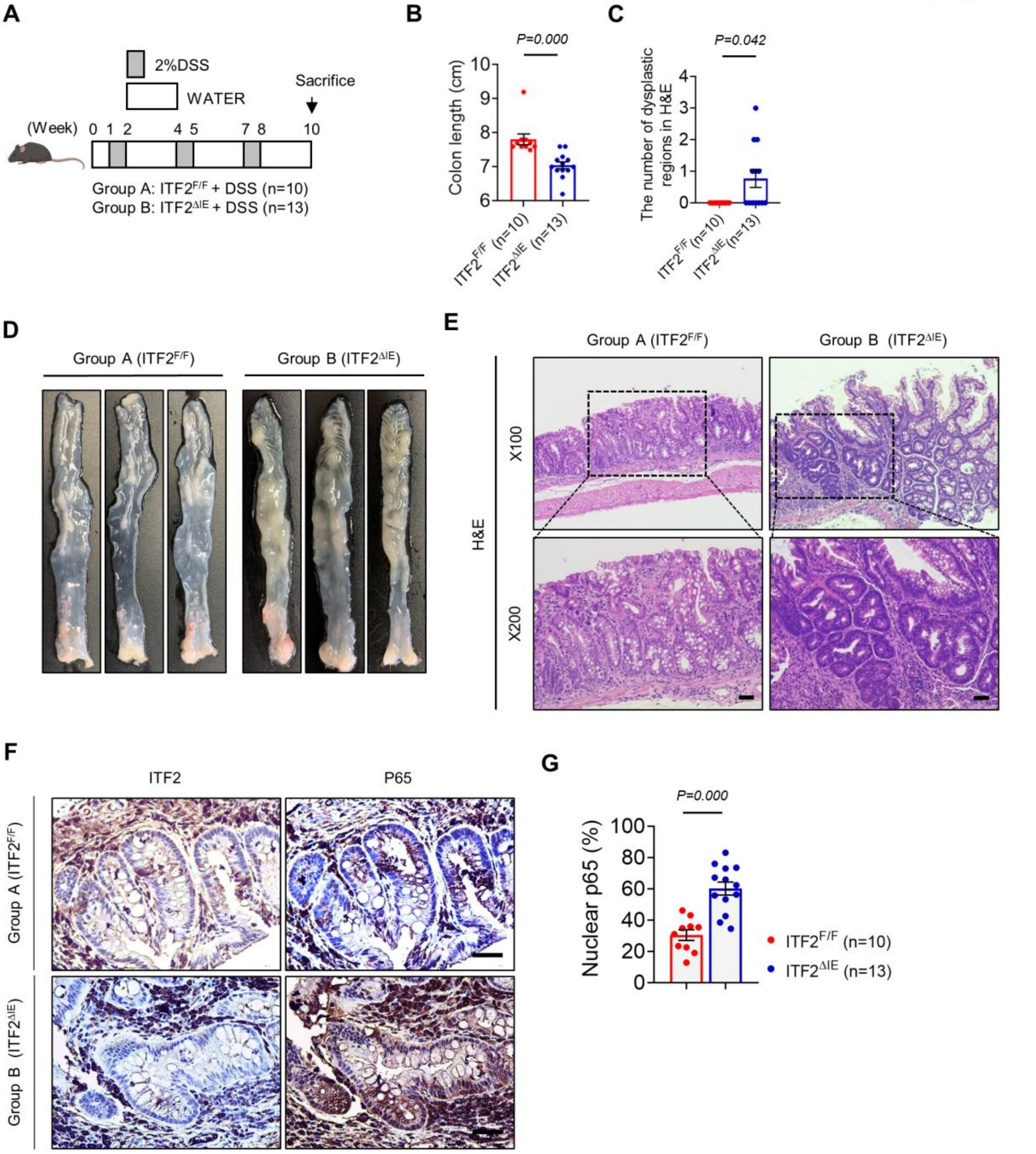

Figure S13. ITF2 loss contributes to tumor formation in DSS-induced colitis mice model. (A) Experimental design. The age-and sex-matched littermates (n = 10) and *ITF2*$^{\Delta IE}$ mice (n = 13) were received three rounds of 2% DSS exposure. At week 10, all mice were sacrificed for further pathological examination. Mouse icon was created with BioRender (BioRender.com). **(B-E)** Colon length **(B)**, dysplastic regions in H&E staining **(C)**, representative colon images **(D)**, and H&E stainings **(E)** in each

group of mice. The data are representative of three independent experiments. **(F)** Representative immunohistochemical images against ITF2 and P65 (The data are representative of three independent experiments.). **(G)** The number of nuclear positive p65 was counted and compared in each group of mice. Scale bars, 100 μm. All results are presented as means ± s.e.m. Statistical significance was determined by the two-tailed Mann–Whitney *U*-test for pairwise comparisons **(B, C, G).** Source data are provided in the Source Data file.

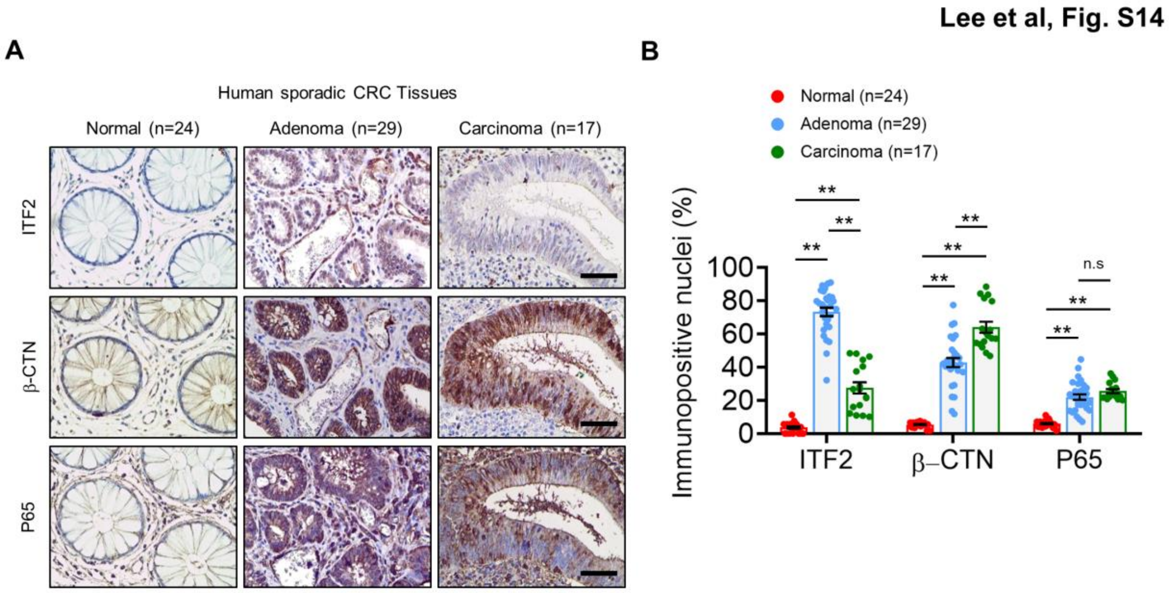

Figure S14. Upregulated β-catenin activation and reciprocally down-regulated ITF2 expression in colon carcinoma. (A) Representative immuno-stained images of ITF2, β-catenin, and p65 in normal, adenoma, and carcinoma in sporadic colon cancer tissues. The data are representative of three independent experiments. **(B)** Nuclear positive expressions of ITF2, β-catenin, and P65 were counted and analyzed in sporadic colon cancer specimens. Scale bars, 100 μm. All results are presented as means ± s.e.m. Statistical significance was determined by Kruskal-Wallis tests followed by the two-tailed Mann–Whitney *U*-test **(B,** $^{**}P < 0.0001$**)** for pairwise comparisons. NS, not significant. Source data are provided in the Source Data file.

Lee et al, Fig. S15

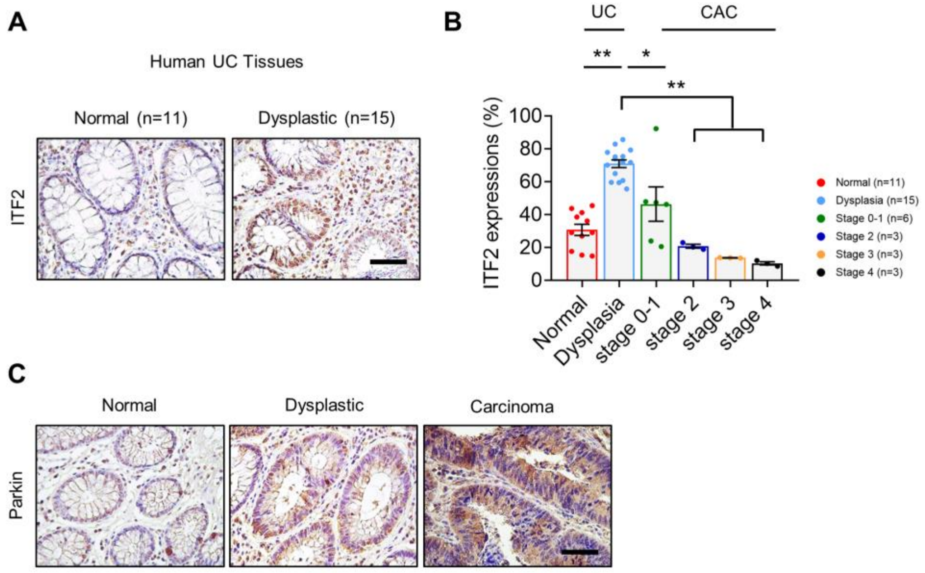

Figure S15. ITF2 is associated with disease stage in CAC patients. (A) Representative immuno-stained images of ITF2 in the patient with UC. The data are representative of three independent experiments. **(B)** Immunostained ITF2 expression levels were calculated by counting stained cells and compared based on the clinical information in both UC and CAC specimens. **(C)** Representative immuno-stained images of Parkin in the UC patient tissues (The data are representative of three independent experiments). Scale bars, 100 μm. All results are presented as means ± SEM. Statistical significance was determined by Kruskal-Wallis tests followed by the two-tailed Mann-Whitney *U* test **(B,** $^{**}P < 0.0001$, $^{*}P = 0.0170$, $^{**}P = 0.0021$**)** for pairwise comparisons. Source data are provided in the Source Data file.

Table S1. List of antibodies used in this study

Antibodies	Cat No.	Clone	Company	Application
**α-tubulin**	sc-53646	10D8	Santa Cruz	WB (1:2000)
**β-tubulin**	sc-9104	H-235	Santa Cruz	WB (1:2000)
**β-catenin**	sc-7963	E-5	Santa Cruz	WB (1:2000), IHC (1:200)
**c-Rel**	sc-6955	B-6	Santa Cruz	WB, IP (1:1000)
**Cleaved caspase-3**	#9664	5A1E	Cell signaling	IHC (1:100)
**CD45**	sc-1178	35-Z6	Santa Cruz	IF (1:100)
**E-cadherin**	#3195	24E10	Cell signaling	IF (1:100)
**Flag-tag**	F3165	M2	Sigma	WB, IP (1:2000)
**HA-tag**	3724S	C29F4	Cell signaling	WB, IP (1:2000)
**His-tag**	PM032		MBL	WB (1:2000)
**IL-6**	ab6672		abcam	IHC (1:100)
**ITF2 (MS)**	sc-101095	367.2	Santa Cruz	IP, WB (1:1000), PLA, IHC (1:100)
**ITF2 (RB)**	sc-366906	H-95	Santa Cruz	IHC (1:200)
**IKB-α**	#4814	L35A5	Cell signaling	WB (1:1000),

				PLA (1:100)
**p-IKB-α**	#2859	14D4	Cell signaling	WB (1:1000)
**Ki-67**	sc-15402	H-300	Santa Cruz	IF (1:500)
**K48-linkage specific poly Ub**	#4289		Cell signaling	WB (1:1000)
**K63-linkage specific poly Ub**	#5621	D7A11	Cell signaling	WB (1:1000)
**Lamin B**	sc-6216	C-20	Santa Cruz	WB (1:100)
**Myc-tag**	#2278	71D10	Cell Signaling	WB, IP (1:2000)
**p65 (RB)**	51-0500		Invitrogen	IHC, PLA (1:100)
**p65 (MS)**	sc-8008	F-6	Santa Cruz	WB, IP (1:1000)
**p65 (RB)**	sc-372	C-20	Santa Cruz	WB (1:1000)
**p-p65 (Ser 536)**	#3033	93H1	Cell signaling	WB (1:1000)
**p50**	sc-8414	E-10	Santa Cruz	WB (1:1000)
**Parkin (RB)**	#2132		Cell signaling	WB (1:1000), IHC (1:100)
**Parkin (MS)**	sc-32282	PRK8	Santa Cruz	IHC (1:100), IP (1:1000)
**Parkin (RB)**	NBP2-67017	JF82-09	Novus	PLA (1:100)
**PCNA**	sc-25280	F-2	Santa Cruz	IF (1:500)
**RelB**	sc-48366	D-4	Santa Cruz	WB, IP (1:1000)

**TNF-α**	ab6671		abcam	IHC (1:100)
**Rabbit IgG HRP**	G21234		Invitrogen	WB (1:5000)
**Mouse IgG HRP**	G21040		Invitrogen	WB (1:5000)
**Goat IgG HRP**	sc-2354		Santa Cruz	WB (1:5000)
**Alexa 488-conjugated rabbit IgG**	A-11008		Invitrogen	IF (1:400)
**Alexa 555-conjugated mouse IgG**	A-21422		Invitrogen	IF (1:400)

WB, western blot; IHC, immunohistochemistry; IF, immunofluorescence; PLA, proximity ligation assay

Table S2. List of reagents used in this study

Antibodies and reagents	Cat No.	Vendor
**AOM**	A5486	Sigma-Aldrich
**Advanced DMEM/F12**	12634-028	Invitrogen
**A/G Agarose beads**	sc-2003	Santa Cruz
**AG490**	S1143	Selleck
**ATP**	A7699	Sigma-Aldrich
**BAY11-7082**	B5556	Sigma-Aldrich
**BAY11-7085**	B5681	Sigma-Aldrich
**B27 supplement**	17504-044	Invitrogen
**Cycloheximide (CHX)**	01810	Sigma-Aldrich
**C-Myc affinity gel**	E6654	Sigma-Aldrich
**C-Myc peptide**	M2435	Sigma-Aldrich

**DSS**	216011080	MPBIO
**Dispase**	17105-041	Invitrogen
**DAPI**	H-1200	Vector
**Ubiquitin activating enzyme E1**	BML-UW9410	Enzo lifesciences
**Recombinant Human UbcH5a/UBE2D1 Protein**	E2-616	Boston Biochem
**EZ-Gel staining solution**	DG-GS1000	DOGEN
**EDTA**	431788	Sigma-Aldrich
**Eosin**	HT110116	Sigma-Aldrich
**Flag affinity gel**	A2220	Sigma-Aldrich
**Glutathione sepharose 4B**	17-0756-01	Sigma-Aldrich
**GlutaMAX-I**	35050-079	Invitrogen
**G 418 disulfate salt**	A1720	Sigma-Aldrich
**HEPES 1M**	15630-056	Invitrogen
**HA affinity gel**	E6779	Sigma-Aldrich
**Hematoxylin**	h-3404	vector
**HA peptide**	I2149	Sigma-Aldrich
**IPTG**	I5502	Sigma-Aldrich
**L-glutathione**	G6529	Sigma-Aldrich
**MK2206**	S1078	Selleck
**Matrigel, GFR, phenol-free**	356231	BD bioscience
**Mouse recombinant noggin**	250-38	Peprotech
**Mouse recombinant EGF**	PMG8041	Invitrogen

**Mouse recombinant R-spondin**	315-32	Peprotech
**Mouse recombinant Wnt-3A**	GF-154	Millipore
**MG132**	C2211	Sigma-Aldrich
**N2 supplement**	17502-048	Invitrogen
**N-Acetylcysteine**	A9165	Sigma-Aldrich
**Ni-NTA agarose**	30210	Qiagen
**Penicillin/Streptomycin**	15140-122	Invitrogen
**Protease inhibitor cocktail (PIC)**	P2714	Sigma-Aldrich
**PMSF**	P7626	Sigma-Aldrich
**PD98059**	#9900	Cell signaling
**Recombinant human TNF-α**	300-01A	Peprotech
**Recombinant murine TNF-α**	315-01A	Peprotech
**Rapamycin**	S1039	Selleck
**SB203580**	#5633	Cell signaling
**Streptavin bead**	17-5113-01	GE healthcare
**TPCA1**	T1452	Sigma-Aldrich
**VectaMount solution**	H-5000	Vector Labs
**3X FLAG peptide**	F4799	Sigma-Aldrich
**70 μm Cell strainer**	352350	BD falcon
**Ubiquitin**	U6253	Sigma-Aldrich

Table S3. The si-RNA sequences used in this study

- **siRNA**

P65:	#1: 5’-AUAUGAGACCUUCAAGAGCAUCATG -3’ and 5’-CAUGAUGCUCUUGAAGGUCUCAUAUGU-3’<br>#2: 5’-CUUUCUACUCUGAACUAAUAAAUCT-3’and 5’-AGAUUUAUUAGUUCAGAGUAGAAAGAG-3’<br>#3: 5’-GCCCAUGGAAUUCCAGUACCUGCCA-3’ and 5’-UGGCAGGUACUGGAAUUCCAUGGGCUC -3’
PRKN	#1: 5’-CAAAGUUAAAGCAUCAGG-3’ and 5’-AAGGAAUAUUCUAGUGAGUUUACUGUC-3’<br>#2: 5’-GAUAGUGUUUGUCAGGUUCAACUCC-3’and 5’-GGAGUUGAACCUGACAAACACUAUCAU-3’<br>#3: 5’-GUCGGAACAUCACUUGCAUUACGTG -3’ and 5’-CACGUAAUGCAAGUGAUGUUCCGACUA -3’
CTNNB1	#1: 5’-CACAACCUUUUAUUACAUCAAGAAG-3’ and 5’-CUUCUUGAUGUAAUAAAAGGUUGUGGA-3’<br>#2: 5’-UGAAUACUGCUACAGCAAUUUCUAA-3’ and 5’-UUAGAAAUUGCUGUAGCAGUAUUCACU -3’<br>#3: 5’-GGAAGCUUCCAGACACGCUAUCATG -3’ and 5’-CAUGAUAGCGUGUCUGGAAGCUUCCUU -3’
RNF6	#1: 5’-GAAUGAGCUUGUUGAGCCAUCAUCA-3’ and 5’-UGAUGAUGGCUCAACAAGCUCAUUCUC-3’<br>#2: 5’-GGGCUAGAAGUAAUGUUACAGUGAG-3’ and 5’-CUCACUGUAACAUUACUUCUAGCCCCA -3’<br>#3: 5’-AUAAUUUACUGAAGCGUCUCUGACA-3’ and 5’-UGUCAGAGACGCUUCAGUAAAUUAUCU -3’
Trim68	#1: 5’-CCUUGCUACAGCAUUGGAACCAACA-3’ and 5’-

	UGUUGGUUCCAAUGCUGUAGCAAGGAC-3’<br>#2: 5’-GAUGAAGUAGAUACAGUCUUCAGGA-3’ and 5’-UCCUGAAGACUGUAUCUACUUCAUCAU -3’<br>#3: 5’-UGAGAGAUUUUACCGCUAUAAUATC-3’ and 5’-GAUAUUAUAGCGGUAAAAUCUCUCAGG -3’

258

259 **Table S4. The qPCR primers used in this study**

hITF2:	F: 5’-TCGGAAGCCTAACTACAGCGA-3’<br>R: 5’-AGATGAGCATTGGCAGCGAG-3’
hIL6:	F: 5’-GTAGCCGCCCCACACAGA-3’<br>R: 5’-CATGTCTCCTTTCTCAGGGCT-3’
IL1B:	F: 5’-GAACAAGTCATCCTCATTGCC-3’<br>R: 5’-CAGCCAATCTTCATTGCTCAAG-3’
hIL8:	F: 5’-ATGACTTCCAAGCTGGCCGTGGCT-3’<br>R: 5’-TCTCAGCCCTCTTCAAAAACTTCTC-3’
hIL23A:	F: 5’-CTCAGGGACAACAGTCAGTTC-3’<br>R: 5’-ACAGGGCTATCAGGGAGCA-3’
hIL12B:	F: 5’-ACCCTGACCATCCAAGTCAAA-3’<br>R: 5’-TTGGCCTCGCATCTTAGAAAG-3’
hIL17A:	F: 5’-CGGACTGTGATGGTCAACCTGA-3’<br>R: 5’-GCACTTTGCCTCCCAGATCACA-3’
hP65:	F: 5′-ATCCCATCTTTGACAATCGTGC-3′<br>R: 5′-CTGGTCCCGTGAAATACACCTC-3′
hRNF6:	F: 5′-TCAGCCTGACTTGAGAGATGG-3′

	R: 5′-TTCGAGTTGCATTTCCTGTG-3′
hTNFA:	F: 5’-GGAGAAGGGTGACCGACTCA-3’<br>R: 5’-CTGCCCAGACTCGGCAA-3’
hTRIM68:	F: 5’-AGGGCCCTGACAACTCTTTT-3’<br>R: 5’-GGGAGCCACAGTCAGTCACATTG-3’
hGAPDH:	F: 5’- GGAGCGAGATCCCTCCAAAAT -3’<br>R: 5’- GGCTGTTGTCATACTTCTCATGG -3’
