# Supplementary material for: Protein stabilization of ITF2 by NF-κB prevents colitis-associated cancer development
Source: Nat Commun. 2023 Apr 25;14:2363. doi: 10.1038/s41467-023-38080-w (PMC10130090; doi:10.1038/s41467-023-38080-w)
Supplement: Supplementary file 3 — Reporting Summary [file 41467_2023_38080_MOESM3_ESM.pdf]

nature portfolio

Corresponding author(s): Hyun-Woo Shin

Last updated by author(s): 03-24-2023

Reporting Summary

Nature Portfolio wishes to improve the reproducibility of the work that we publish. This form provides structure for consistency and transparency in reporting. For further information on Nature Portfolio policies, see our Editorial Policies and the Editorial Policy Checklist.

Statistics

For all statistical analyses, confirm that the following items are present in the figure legend, table legend, main text, or Methods section.

n/a	Confirmed	
☐	☒	The exact sample size (*n*) for each experimental group/condition, given as a discrete number and unit of measurement
☐	☒	A statement on whether measurements were taken from distinct samples or whether the same sample was measured repeatedly
☐	☒	The statistical test(s) used AND whether they are one- or two-sided<br>*Only common tests should be described solely by name; describe more complex techniques in the Methods section.*
☒	☐	A description of all covariates tested
☐	☒	A description of any assumptions or corrections, such as tests of normality and adjustment for multiple comparisons
☐	☒	A full description of the statistical parameters including central tendency (e.g. means) or other basic estimates (e.g. regression coefficient) AND variation (e.g. standard deviation) or associated estimates of uncertainty (e.g. confidence intervals)
☐	☒	For null hypothesis testing, the test statistic (e.g. *F*, *t*, *r*) with confidence intervals, effect sizes, degrees of freedom and *P* value noted<br>*Give P values as exact values whenever suitable.*
☒	☐	For Bayesian analysis, information on the choice of priors and Markov chain Monte Carlo settings
☒	☐	For hierarchical and complex designs, identification of the appropriate level for tests and full reporting of outcomes
☐	☒	Estimates of effect sizes (e.g. Cohen's *d*, Pearson's *r*), indicating how they were calculated

Our web collection on statistics for biologists contains articles on many of the points above.

Software and code

Policy information about availability of computer code

Data collection	ImageJ v.1.53k<br>ZEN v3.2<br>BioRad CFX Maestro v4.1.2433.1219<br>BD FACSDiva v8.0.1<br>SkanIt RE v5.0
Data analysis	Statistical analysis : SPSS v25.0<br>Illustrative figures : GraphPad Prism v 8.3.1, SigmaPlot v10.0<br>Prediction of 3D protein structure: AlphaFold v2.0

For manuscripts utilizing custom algorithms or software that are central to the research but not yet described in published literature, software must be made available to editors and reviewers. We strongly encourage code deposition in a community repository (e.g. GitHub). See the Nature Portfolio guidelines for submitting code & software for further information.

Data

Policy information about availability of data

All manuscripts must include a data availability statement. This statement should provide the following information, where applicable:

- Accession codes, unique identifiers, or web links for publicly available datasets
- A description of any restrictions on data availability
- For clinical datasets or third party data, please ensure that the statement adheres to our policy

Publicly available datasets (GSE38713, GSE11223, GSE37283, GSE112366, GSE75916, GSE1710, GSE20881, GSE87473, GSE3629) were retrieved from Gene Expression Omnibus (https://www.ncbi.nlm.nih.gov). All data relevant to the study are included in the article and supplementary information.

Human research participants

Policy information about studies involving human research participants and Sex and Gender in Research.

Reporting on sex and gender	Human CAC tissue specimens and sporadic CRC specimens were obtained from the Seoul National University Hospital Tissue Bank and Boramae Hospital Tissue Bank.<br>CAC patients: Among 22 CAC samples, Male (16) and Female (6).<br>UC patients: Among 15 UC samples, Male (10), Female (5).<br>CRC patients: Among 29 CRC samples, Male (17) and Female (12).
Population characteristics	Ulcerative colitis (UC) subjects age distribution: between 19 and 72.<br>Sporadic CRC patient subjects age distribution: between 29 and 80.<br>Colitis associated cancer (CAC) subjects age distribution : between 31 and 76<br>A pathologist (J.H.K.) reviewed all slides from all cases to check for evidence of colitis and carcinoma, and a gastroenterologist (S.J.K.) reviewed the medical records for the clinical history of IBD. They then selected the proper tissue blocks for further analysis, and samples were classified into three groups: normal, dysplasia, and carcinoma.
Recruitment	Human CAC tissue specimens and sporadic CRC specimens were obtained from the Seoul National University Hospital Tissue Bank and Boramae Hospital Tissue Bank. Patient's medical records were reviewed, and tissues from all potential cases of CAC and CRC were collected.
Ethics oversight	All procedures were approved by the Institutional Review Board and Committee of Seoul National University Hospital (approval 1912-056-1087 and 2105-055-1218).

Note that full information on the approval of the study protocol must also be provided in the manuscript.

Field-specific reporting

Please select the one below that is the best fit for your research. If you are not sure, read the appropriate sections before making your selection.

☒ Life sciences ☐ Behavioural & social sciences ☐ Ecological, evolutionary & environmental sciences

For a reference copy of the document with all sections, see nature.com/documents/nr-reporting-summary-flat.pdf

Life sciences study design

All studies must disclose on these points even when the disclosure is negative.

Sample size	Sample sizes are set to 3 or more based on the number of replicates necessary to achieve statistical significance derived from previous studies and our experiences (PMID: 26342525, PMID: 33957165, PMID: 30804419)
Data exclusions	No data were excluded from the analysis.
Replication	Results were consistently replicated across experiments as indicated in figure legends.
Randomization	Mice were assigned randomly to experimental and control groups. Other experiments were not relevant as there were not grouping used in this study.
Blinding	Two examiners blinded to the experimental group determined and averaged the expression levels. When the examiners disagreed, a consensus was reached by reviewing the specimens at a multi-head microscope by our research team.

Reporting for specific materials, systems and methods

We require information from authors about some types of materials, experimental systems and methods used in many studies. Here, indicate whether each material, system or method listed is relevant to your study. If you are not sure if a list item applies to your research, read the appropriate section before selecting a response.

Materials & experimental systems

n/a	Involved in the study
☐	☒ Antibodies
☐	☒ Eukaryotic cell lines
☒	☐ Palaeontology and archaeology
☐	☒ Animals and other organisms
☒	☐ Clinical data
☒	☐ Dual use research of concern

Methods

n/a	Involved in the study
☒	☐ ChIP-seq
☐	☒ Flow cytometry
☒	☐ MRI-based neuroimaging

Antibodies

Antibodies used

All antibodies used in the study have been listed in supplementary data Table S1. Briefly;
Antibodies; Catalog number; Company; Applications
a-tubulin; sc-53646; Santa Cruz; WB (1:2000)
b-tubulin; sc-9104; Santa Cruz; WB (1:2000)
b-catenin; sc-7963; Santa Cruz; WB (1:2000), IHC (1:200)
c-Rel; sc-6955; Santa Cruz; WB, IP (1:1000)
Cleaved caspase-3; #9664; Cell signaling; IHC (1:100)
CD45; sc-1178; Santa Cruz; IF (1:100)
E-cadherin; #3195; Cell signaling; IF (1:100)
Flag-tag; F3165; Sigma; WB, IP (1:2000)
HA-tag; 3724S; Cell signaling; WB, IP (1:2000)
His-tag; PM032; MBL; WB (1:2000)
IL-6; ab6672; abcam; IHC (1:100)
ITF2 (MS); sc-101095; Santa Cruz; IP, WB (1:1000), PLA, IHC (1:100)
ITF2 (RB); sc-366906; Santa Cruz; IHC (1:200)
IKB-a; #4814; Cell signaling; WB (1:1000), PLA (1:100)
p-IKB-a; #2859; Cell signaling; WB (1:1000)
Ki-67; sc-15402; Santa Cruz; IF (1:500)
K48-linkage specific poly Ub; #4289; Cell signaling; WB (1:1000)
K63-linkage specific poly Ub; #5621; Cell signaling; WB (1:1000)
Lamin B; sc-6216; Santa Cruz; WB (1:100)
Myc-tag; #2278; Cell Signaling; WB, IP (1:2000)
p65 (RB); 51-0500; Invitrogen; IHC, PLA (1:100)
p65 (MS); sc-8008; Santa Cruz; WB, IP (1:1000)
p65 (RB); sc-372; Santa Cruz; WB (1:1000)
p-p65 (Ser 536); #3033; Cell signaling; WB (1:1000)
p50; sc-8414; Santa Cruz; WB (1:1000)
Parkin (RB); #2132; Cell signaling; WB (1:1000), IHC (1:100)
Parkin (MS); sc-32282; Santa Cruz; IHC (1:100), IP (1:1000)
Parkin (RB); NBP2-67017; Novus; PLA (1:100)
PCNA; sc-25280; Santa Cruz; IF (1:500)
RelB; sc-48366; Santa Cruz; WB, IP (1:1000)
TNF-a; ab6671; abcam; IHC (1:100)
Rabbit IgG HRP; G21234; Invitrogen; WB (1:5000)
Mouse IgG HRP; G21040; Invitrogen; WB (1:5000)
Goat IgG HRP; sc-2354; Santa Cruz; WB (1:5000)
Alexa 488-conjugated rabbit IgG; A-11008; Invitrogen; IF (1:400)
Alexa 555-conjugated mouse IgG; A-21422; Invitrogen; IF (1:400)

Validation

All primary antibodies were confirmed on the species and application through the validation statement on the manufacturer's website and their use in the literature.
Antibodies; Catalog number; Species reactivity; Clone; Applications;
a-tubulin; sc-53646; broad species origin; 10D8; WB, IP, IF, ELISA product citations (159)
b-tubulin; sc-9104; ; H-235; ; product citations (319)
b-catenin; sc-7963; mouse, rat, human; E-5; WB, IP, IF, IHC(P), ELISA; product citations (921)
c-Rel; sc-6955; mouse, rat, human; B-6; WB, IP, IF, IHC(P); product citations (127)
Cleaved caspase-3; #9664; mouse, rat, human, monkey; 5A1E; WB, IP, IHC, IF, F; product citations (9224)
CD45; sc-1178; mouse, rat, human; 35-Z6; WB, IP, IF, IHC(P), FCM; product citations (82)
E-cadherin; #3195; mouse, human; 24E10; WB, IHC, IF, F; product citations (4154)
Flag-tag; F3165; all; M2; WB, IP, ICC, IF, ELISA, EIA, ChIP, F; product citations (7577)
HA-tag; #3724; all; C29F4; WB, IP, IF, IHC, ChIP, F; product citations (2244)
His-tag; PM032; ; ; WB, IP, IC; product citations (13)
IL-6; ab6672; human; ; WB, IHC(P); product citations (507)
ITF2 (MS); sc-101095; mouse, rat, human; 367.2; WB, IP, ELISA; product citations (2)
ITF2 (RB); sc-366906; mouse, rat, human; H-95; WB, IP, IF, ELISA; product citations ()
IKB-a; #4814; mouse, rat, human, monkey, bovine, pig; L35A5; WB, IP, IHC, IF, F; product citations (1053)

p-IKB-a; #2859; mouse, rat, human, monkey; 14D4; WB, IP; product citations (1420)
Ki-67; sc-15402; ; H-300; ; product citations (48)
K48-linkage specific poly Ub; #4289; all; ; WB; product citations (84)
K63-linkage specific poly Ub; #5621; all; D7A11; WB; product citations (168)
Lamin B; sc-6216; ; C-20; ; product citations (210)
Myc-tag; #2278; all; 71D10; WB, IP, IF, F; product citations (619)
p65 (RB); 51-0500; human, mouse, non-human primate; ; WB, IHC, IF, ELISA; product citations (14)
p65 (MS); sc-8008; mouse, rat, human; F-6; WB, IP, IF, IHC(P), FCM, ELISA; product citations (2129)
p65 (RB); sc-372; ; C-20; ; product citations (1007)
p-p65 (Ser 536); #3033; mouse, rat, human, monkey, hamster, pig; 93H1; WB, IP, IF, F; product citations (5521)
p50; sc-8414; mouse, rat, human; E-10; WB, IP, IF, IHC(P), FCM, ELISA; product citations (345)
Parkin (RB); #2132; mouse, rat, human; ; WB; product citations (127)
Parkin (MS); sc-32282; mouse, rat, human; PRK8; WB, IP, IF, IHC(P), FCM; product citations (265)
Parkin (RB); NBP2-67017; mouse, rat, human; JF82-09; WB, IP, ICC/IF, IHC(P), F; product citations (5)
PCNA; sc-25280; mouse, rat, human; F-2; WB, IP, IF, IHC(P), ELISA; product citations (370)
RelB; sc-48366; mouse, rat, human; D-4; WB, IP, IF, IHC(P), ELISA; product citations (31)
TNF-a; ab6671; human, cynomolgus monkey; ; WB, ICC/IF, IHC(P), ELISA; product citations (641)
Rabbit IgG HRP; G21234; rabbit; ; WB, IP, IHC, ELISA; product citations (792)
Mouse IgG HRP; G21040; mouse; ; WB, IHC, ELISA; product citations (701)
Goat IgG HRP; sc-2354; goat; ; WB, IHC; product citations (595)
Alexa 488-conjugated rabbit IgG; A-11008; rabbit; ; IHC, ICC/IF, F; product citations (7841)
Alexa 555-conjugated mouse IgG; A-21422; mouse; ; IHC, ICC/IF, F; product citations (926)

Eukaryotic cell lines

Policy information about cell lines and Sex and Gender in Research

Cell line source(s)

Human embryonic kidney (HEK293T), and human colon cancer cell lines (Colo320DM, SW480, HCT116, LoVo, DLD-1, LS174T, HT-29, SNU-283, HCT-15, SNU-1033, CaCo2, WiDr, and Colo205) were obtained from the American Type Culture Collection (VA, USA) and/or Korean Cell Line Bank (Seoul, South Korea).
Cell; Catalog number; Company; Organism; Morphology; Origin; Disease
293T; CRL-3216; ATCC; Human; epithelial; kidney;
Colo320DM; 10220; KCLB; Human; rounded and refractile; Colon; Adenocarcinoma
Colo320HSR; 10220.1; KCLB; Human; rounded and refractile; Colon; Adenocarcinoma
SW480; 10228; KCLB; Human; epithelial; Colon; Adenocarcinoma
HCT116; 10247; KCLB; Human; epithelial; Colon; Carcinoma
LoVo; 10229; KCLB; Human; epithelial; Colon; Adenocarcinoma
DLD-1; 10221; KCLB; Human; epithelial; Colon; Adenocarcinoma
LS174T; 10188; KCLB; Human; epithelial; Colon; Adenocarcinoma
HT-29; 30038; KCLB; Human; epithelial; Colon; Adenocarcinoma
SNU-283; 00283; KCLB; Human; epithelial; Colon; carcinoma
HCT-15; 10225; KCLB; Human; epithelial; Colon; Adenocarcinoma
SNU-1033; 01033; KCLB; Human; epithelial; Colon; carcinoma
Caco-2; 30037.1; KCLB; Human; epithelial-like; Colon; Colorectal Adenocarcinoma
WiDr; 10218; KCLB; Human; epithelial; Colon; Adenocarcinoma
Colo205; 10222; KCLB; Human; epithelial; Colon; Adenocarcinoma
HeLa; 10002; KCLB; Human; epithelial; Cervix; Adenocarcinoma

Authentication

The cell lines were authenticated by the supplier based on growth, morphology, and STR DNA profiling of the cell line was analyzed in Korean Cell Line Bank (Seoul, South Korea).

Mycoplasma contamination

Mycoplasma contamination was routinely tested when cell growth or shape was changed. Cell lines tested negative for Mycoplasma.

Commonly misidentified lines
(See ICLAC register)

No commonly misidentified cell lines were used in the study

Animals and other research organisms

Policy information about studies involving animals; ARRIVE guidelines recommended for reporting animal research, and Sex and Gender in Research

Laboratory animals

Wild-type mice were utilized (Koatech, Gyeonggi-do, South Korea). In addition, Genetically engineered C57BL/6 mice were used. Both male and female mice aged 6-8 weeks were used at the time of experiments unless indicated otherwise. ITF2fl/fl mice were produced by ToolGen Inc (Seoul, South Korea). CRISPR/Cas9 were designed to target the intron 1 and 18 of the ITF2 gene, respectively. The synthesized single-stranded oligonucleotides (ssDNAs) were co-injected into fertilized wild-type oocytes together with Cas9 protein and guide RNA (gRNAs). To screen founders carrying both LoxP sites in the ITF2 gene, we performed PCR assay using genomic DNA derived from pubs that were generated from the microinjected embryos. The genomic regions spanning each LoxP site were amplified by PCR. The candidates were cloned in T-Blunt PCR Cloning Vector (Solgent Co. Ltd., Daejeon, South Korea), and were validated by direct sequencing analysis. All mice maintained in the specific pathogen-free (SPF) room under controlled temperature (23 ± 3℃),and humidity (40-60%) conditions with 12/12h light/ dark cycle. The center has a system that can ventilate 15-16 times/ hour with an airflow rate of 13-18 cm/ sec.

Wild animals	No wild animals were used in the study.
Reporting on sex	Both male and female mice aged 6-8 weeks were used at the time of experiments unless indicated otherwise. Sample sizes for mouse experiments were empirically determined, and mice were randomly allocated to the control or experimental groups. Animal studies were conducted in a gender- and age-matched manner using littermates for each experiment. All mice were of C57/BL6 background.
Field-collected samples	No field collected samples were used in the study.
Ethics oversight	All in vivo experiments were approved by the Institutional Animal Care and Use Committee (IACUC) of Seoul National University (approval SNU-180306-3-2, SNU-180306-2-3, and SNU-181214-4-3) and carried out in strict accordance with good animal practice as defined by the governmental and international guidelines of animal experiments.

Note that full information on the approval of the study protocol must also be provided in the manuscript.

Flow Cytometry

Plots

Confirm that:

☒ The axis labels state the marker and fluorochrome used (e.g. CD4-FITC).

☒ The axis scales are clearly visible. Include numbers along axes only for bottom left plot of group (a 'group' is an analysis of identical markers).

☒ All plots are contour plots with outliers or pseudocolor plots.

☒ A numerical value for number of cells or percentage (with statistics) is provided.

Methodology

Sample preparation	Colo320DM cells were transfected with Myc-ITF2 followed by the TNF stimulation for 8 h, and 24 h respectively. The cells were resuspended in binding buffer and then stained with FITC-conjugated annexin V antibody for 15minutes in the dark at room temperature, and then incubated with propidium iodide (PI) sequentially.
Instrument	BD FACSCanto II
Software	BD FACSDiva v8.0.1
Cell population abundance	We shown as percentage of live, apoptotic and necrotic cells
Gating strategy	The cells were stained with annexin V and PI. The viable cells were located in the lower left quadrant (annexin V -/PI -). Early apoptotic and necrotic cells were sorted in the lower right (annexin V +/PI -) and the upper left (annexin V -/PI +) quadrant, respectively. Late apoptotic cells were located in the upper right quadrant (annexin V +/PI +) .

☒ Tick this box to confirm that a figure exemplifying the gating strategy is provided in the Supplementary Information.
